# Teaching DNP Students Evidence-Based Practice and Quality Improvement

**DOI:** 10.1097/AJN.0000000000000236

**Published:** 2026-01-22

**Authors:** Julee Waldrop, Kerry A. Milner, Staci S. Reynolds, Ellen Fineout-Overholt, Jayne Jennings Dunlap

**Affiliations:** **Julee Waldrop** is professor emeritus at the University of North Carolina at Chapel Hill School of Nursing and clinical associate at the Duke University School of Nursing, Durham, NC. **Kerry A. Milner** is adjunct professor of nursing at Sacred Heart University, Fairfield, CT. **Staci S. Reynolds** is clinical professor at the Duke University School of Nursing. **Ellen Fineout-Overholt** is a consultant and coach at Thought Transformation for Life, Hallsville, TX. **Jayne Jennings Dunlap** is associate professor at the Texas Woman's University College of Nursing in Houston. Contact author: Julee Waldrop, julee.waldrop@duke.edu. The authors have disclosed no potential conflicts of interest, financial or otherwise.

## Abstract

It's time to eliminate inaccurate language and clarify competencies.

The DNP degree is now 20 years old. Endorsed in 2004 by the American Association of Colleges of Nursing (AACN) as the terminal practice degree for nurses, the DNP was designed to prepare expert clinicians and leaders who could bridge the gap between research and practice.^1^ However, we have observed over the years the continuing struggle to produce graduates with consistent competencies. The core issue? Most DNP programs are teaching students to conduct small research studies when they should be teaching evidence-based practice (EBP)—applying the best available evidence to patient care decisions—and quality improvement (QI)—systematic methods to improve health care processes and outcomes. Most leaders and faculty teaching in DNP programs would say they are doing the best they can with what they know and what they have. However, there is a better way to fulfill the degree as it was originally intended.^2^ Equipping faculty with knowledge of true practice change methods is imperative, rather than having them teach the use of unsuitable research methods for DNP projects. If we embrace both EBP and QI, the nursing profession—from bedside to leadership—can achieve the sustainable improvement in patient care and health care quality that the DNP degree was meant to deliver.

**EBP *and* QI**. The better way forward is combining EBP *and* QI to support evidence-based decision-making at every level, from point of care to executive leadership.^3^ DNP graduates should not learn to conduct poorly designed, unsustainable research studies. They must learn to be experts in EBP and QI. We are united in our determination to refocus attention on the intended purpose of the DNP graduate: a leader skilled in both EBP and QI, not one or the other. Although different models have been developed and various leaders have their preferences, we agree on this major point: we must review how DNP curricula are developed and taught, along with the required project outcomes for every program. We invite the American Nurses Association, the AACN, and all nursing organizations with an interest in nursing education to join us in creating reform to ensure that DNP projects use combined EBP and QI methods—methods distinctly different from research—and include these critical steps:

1. Understand the clinical problem using internal and external data.
2. Develop an evidence-searching question that reflects the problem, guides an unbiased search for evidence, and defines key search terms.
3. Conduct an evidence search, critical appraisal, and synthesis to develop practice recommendations.
4. Create project aims that include process, balancing, and outcome measures.
5. Implement evidence-based recommendations using relevant frameworks.
6. Complete repeated small tests of practice change (interventions) during implementation using QI methods, with all aspects described in enough detail to be transferable.
7. Evaluate project results using data monitoring and visualization with run charts or statistical process control charts to identify sustainable improvements over time.
8. Disseminate these endeavors to appropriate groups for sustainable impact.

Applying the above steps is what the proper use of focused DNP skills should look like and will make sustainable impact more likely. Data analyzed using QI methods—not statistical significance testing—support sustainable practice change. Research methods and language have no place in this paradigm.

**Self-assessment**. The first step in changing any behavior is to become aware of it. One place to start increasing one's awareness is to observe how, as faculty in a DNP program, we talk and write about what we and our students are doing. As a profession, we need to recognize that research terms have been entrenched for many years in both nursing education and practice, while EBP and QI paradigms are newer and likely were not part of many faculty members' educational programs.

Another opportunity for self-reflection and growth is to notice when we or others use inaccurate language regarding evidence-based decision-making. For example, a student may mention they plan to “recruit enough subjects for their QI project in order to determine statistical significance.” This presents a great opportunity to teach proper EBP and QI evaluation, but doing so depends on our understanding of the aspects of the students' statement that are inaccurate. Table 1 (see http://links.lww.com/AJN/A298) provides common language confusions to encourage reflection on our understanding. These common language confusions reveal deeper misunderstandings about the difference between research and EBP/QI. For instance, patients receiving an EBP change are not “subjects” or a “sample”—they are the entire population of patients who should receive best practice. In research, we recruit subjects to generate generalizable knowledge; in EBP/QI, we implement best practices with all eligible patients as part of usual care. For example, evidence supports the recommendation that all patients who score above 10 on the Patient Health Questionnaire-9 receive a referral to behavioral health services. Therefore, all patients who meet this evidence-based requirement receive a referral.

Similarly, EBP/QI projects don't test for statistical significance in pursuit of generalizable results (this is the province of research), but are focused on clinically meaningful outcomes that are locally applicable, using frequent, iterative tests of change to implement practice changes (for example, Plan–Do–Study–Act [PDSA] cycles). Data are collected often (weekly, monthly) to evaluate sustained impact, which is almost exclusively followed through the use of run charts or control charts. Control charts plot data at set intervals to allow teams to see changes in outcomes or processes over time, so they can make iterative changes to their practice during appropriate intervals, such as the “study” phase of PDSA cycles.

Typical DNP projects are often small, local, quasi-experimental research studies that do not equip DNP graduates to take their place as leaders of quality health care.^4^ These projects take a toll on practice partners and health care organizations, because they are not focused on sustainable practice improvement.^5^,^6^ They also burden journal editors who receive manuscripts reporting findings from these small-sample studies. Having DNP students complete small research studies perpetuates confusion among our health care partners about what to expect from DNP graduates (who should be experts in EBP and QI) and undermines the value of the DNP degree and its graduates.^7^,^8^ The DNP-prepared nurse must have a unique and distinctly different skill set in EBP/QI.

**Need for faculty development**. Given the frequent lack of clarity about appropriate DNP projects and language,^4^ faculty in DNP and PhD programs require development in these areas. EBP/QI content is not regularly included in the curricular content in PhD programs. Unfortunately, the same is true for many DNP programs when students are taught by nurses prepared with a PhD. In this way, some DNP graduates may become faculty who perpetuate this problem. Faculty development for all those who teach in DNP and PhD programs, including librarians and statisticians, can make a difference not only in how DNP projects are conducted but also in scholarship opportunities.^9^,^10^ Many nursing journals now accept manuscripts on EBP and QI initiatives.

If faculty learn the difference between research methods and EBP/QI methods and use the latter model to drive their projects,^11^-^13^ they will begin to see their graduates lead in sustainable practice improvement. Seeing research be put into practice and become part of sustainable practice change can provide nurse faculty with a sense of purpose and pride that they are contributing to making a difference that is visible, measured, and sustainable.

There are many resources available for faculty to use to begin to enhance their self-study of EBP and QI, and we hope you will take advantage of them (see Table 2 at http://links.lww.com/AJN/A299).

**Now is the time for change**. We urge readers to heed our call and be alert to inaccuracies in DNP projects and the language we use to describe them, striving to put into practice the content and competencies that lead to the best outcomes.

Faculty are uniquely positioned to pass this approach on to future faculty. Unfortunately, we have encountered well-intended faculty across various institutions who, despite incorrect approaches, believe they are already teaching EBP and QI and therefore do not need faculty development. This may be the greatest challenge of all. Imagine the positive momentum we could achieve together if we summoned our collective courage to learn from our mistakes and work toward a new understanding with an open mind. We can make a sustainable health care system impact as lifelong learners open to exploring the best paradigm for producing future nursing leaders.
